# Neonatal Septicaemia

**Published:** 1965-10

**Authors:** O. C. Lloyd


### NEONATAL SEPTICAEMIA DUE TO PSEUDOMONAS AERUGINOSA (PYOCYANEA)

*A Clinico-Pathological Conference held in the University of Bristol on 16th February 1965*

CHAIRMAN: DR. O. C. LLOYD

*Dr. Beryl Corner:* The mother of this patient was admitted to Bristol Maternity Hospital 26 hours before delivery with ruptured membranes. Labour was normal, ending with a spontaneous vertex delivery of a male child which looked immature and weighed 4 lb 12 oz. His general behaviour, appearance, and head circumference which was just over 13 inches, suggested that the gestation was probably about 36 weeks. He breathed spontaneously and there was no problem for the first few hours. Bottle feeding started quite satisfactorily at 12 hours.

At about 18 hours he became lethargic and irritable with a shrill cry, increasing generalized oedema, slight jaundice and refusal to suck. Then quite suddenly at 30 hours he became very pale, stopped breathing, and was resuscitated with oxygen. Following this he remained very pale and in what was described as "a shocked condition", but was stiff with a full fontanelle. An intracranial catastrophe was suspected, possibly haemorrhage or meningitis. An intravenous drip of 5 per cent dextrose in water was given through the umbilical vein to combat the shocked condition, and investigations were carried out. In view of the possibility of an overwhelming infection blood for culture, various swabs, urine and C.S.F. were examined.

During the second day a rather unusual rash appeared on the trunk; the general condition was very poor, with sclerema, that is generalized thickening and oedema of subcutaneous tissues. The abdomen was somewhat distended and on the skin there was an erythematous blotchy rash which developed a vesicular appearance. This was considered to be an infective rash, and different from neonatal urticaria which we commonly see in newborn babies. Streptomycin intramuscularly was given and lumbar puncture was performed but the C.S.F. was blood-stained. A number of bacteriological and biochemical investigations were performed, and a chest X-ray which showed no significant abnormality. As the child was considered to be in a failing condition treatment with digoxin and intravenous hydrocortisone was given about 2–3 hours before death, which occurred at 48 hours following gradual respiratory failure with gasping and irregular respiration. Our diagnosis was an overwhelming infection, i.e. septicaemia, possibly with meningitis, but the baby died too soon for us to get the results of any of the investigations.

I would add that 24 hours after delivery the mother developed a temperature of over 101°F, which rose after 48 hours and was diagnosed as due to streptococcal infection (a haemolytic streptococcus, Lancefield Group A, was grown from a high vaginal swab) and responded very quickly to antibiotic treatment. No other organisms were grown from her swabs.

This was all the information available when the baby was alive: a good deal more became evident at post mortem, and in retrospect this child indeed was shown to have an infection, which occurred at the onset of an epidemic in the premature baby unit at the maternity hospital. Perhaps I should pass the story on now to Professor Gillespie.

*Professor W. A. Gillespie:* This diagnosis was correct. It was an overwhelming infection. A blood culture was taken and the cerebrospinal fluid was also cultured and although it did not show much increase in cells, *Pseudomonas aeruginosa* (*pyocyanea*) grew from the cerebrospinal fluid and from the blood. Swabs around the umbilicus also grew the Pseudomonas which was resistant to the common antibiotics except polymixin and colistin. I think that is all I can say, except on the epidemiological aspect.

*Dr. O. C. Lloyd:* Here we have, in sum, a baby who was not very big at birth and who started getting ill within 24 hours. It suffered an acute overwhelming infection of an organism which is not commonly found in septicaemia.

*Professor Gillespie:* Not very uncommonly in certain kinds of patient.

*Dr. Lloyd:* It was present but not particularly suspected in this child, and the streptococcus which was found from the mother's throat was a "red herring". Is that right?

*Dr. Corner:* As far as the baby was concerned. The mother quite obviously had an infection of the genital tract as a streptococcus was grown from a high vaginal swab.

*Professor Gillespie:* Was it an anaerobic one?

*Dr. Corner:* No. It was an ordinary group A streptococcus. She had her tonsils out as a child, and had no history of tonsillitis. This infant of course had no fever throughout.

*Professor A. V. Neale:* May I ask Dr. Corner more about the rash. Erythematous, I think you said, or was it purpuric?

*Dr. Corner:* Yes. It came out in little patches as an erythema, with isolated red spots on the abdominal wall, which then developed vesicles. We had no doubt that the rash was an infective one.

*Professor Neale:* Did you take a swab from the region?

*Dr. Corner:* Yes, we did have swabs from the armpit, from every orifice, the abdominal skin, and every other usual area for swabbing.

*Dr. Lloyd:* For which the Path. Department were duly grateful.

*Dr. Corner:* We always swab the nose, umbilicus, skin lesions, and examine urine and faeces when infection is suspected.

*Dr. Lloyd:* Would the white cell count have been of value?

*Dr. Corner:* It would have been of no value at all. A newborn infant has a leucocytosis with a white cell count of 20–24,000/cu mm normally.

*Professor Neale:* Dr. Corner, did you think there was a pneumonic condition of the left lung?

*Dr. Corner:* Well, we wondered whether this child had got a respiratory infection and congenital pneumonia, remembering that the membranes ruptured 26 hours before delivery, so there was time for it to develop. Of course we did not know the results of the mother's swab until after the child had died, so that really might have proved a "red herring".

*Professor Neale:* Did Dr. Corner find any clinical evidence suggesting meningitis?

*Dr. Corner:* Yes. The C.S.F. culture gave a pure growth of Pseudomonas and the blood culture also.

*Professor Neale:* Any cells in the C.S.F.?

*Dr. Corner:* I do not think there was a cell count, as the fluid was very blood-stained, thought to be due to trauma from the lumbar puncture.

*Professor Neale:* Again, just for the record, was the fontanelle bulging?

*Dr. Corner:* It was tense, but not bulging. The baby was very sick, very irritable, and with an irritable squeaky cry. It refused to suck.

*Dr. Lloyd:* How long does the fontanelle have to be tense before it starts bulging?

*Professor Neale:* It varies with the person who palpates it.

*Dr. Lloyd:* I know it feels tense, but presumably in order to feel it bulge, it has to be under tension long enough for the fibrous tissue to stretch.

*A paediatrician:* I would have thought the fontanelle is a highly tricky sign, it is sometimes tense very quickly and sometimes with a raging meningitis it is not tense. It is the sort of sign that when it is positive, helps, and when it is negative it does not.

*Dr. Lloyd:* I think it is time we asked the pathologist what he found.

*Dr. J. Harbison:* I carried out the post-mortem examination on this infant 9 hours after death. It was a normally developed male infant with a red patchy rash over the anterior surface of the limbs and trunk. There was cyanosis of the nail beds. The main features I found inside the baby were as follows:

Both lungs contained abscesses, the largest (1 cm in diameter) in the posterior basal segment of the left lower lobe; another, slightly smaller, on the diaphragmatic surface of the right lung. The abscesses were surrounded by a narrow zone of haemorrhage. Microscopically these were multiple, and scattered throughout both lower lobes. It is of interest to note that the inflammatory reaction in them consisted of macrophages, lymphocytes and some eosinophils; neutrophils were virtually absent. Gram stains revealed only chains of Gram-positive cocci, but no Gram-negative bacilli. A swab from one of these abscesses however grew both Group A haemolytic streptoccoci (Griffith type M12, R–, T12) and *Pseudomonas aeruginosa*.

The liver was congested and close scrutiny of its peritoneal and cut surfaces revealed many small pinhead-sized white patches. Microscopically these proved to be foci of necrosis, quite random in their distribution. It is noteworthy that there was no inflammatory reaction around them. Although they therefore were not strictly speaking abscesses, a Gram stain showed Gram-negative bacilli in the necrotic material. A

reticulin stain did not demonstrate any loss of architecture in the necrotic areas, indicating that these occurred a day or two before death. The round cell infiltrations scattered throughout the liver were areas of extra-medullary haemopoiesis.

Both kidneys to the naked eye were found to have petechial haemorrhages in the cortices. Histological examination revealed in addition small necrotic foci in the cortex similar to those in the liver, but smaller and fewer in number. They also contained Gram-negative bacilli. No such lesions were seen in the spleen.

I did not find any evidence of meningitis either naked eye or microscopically. There was a small subarachnoid haemorrhage beneath the cerebellum. It probably dated from the child's birth. A section of the ligamentum teres of the liver did not demonstrate any sign of sepsis there, which excluded direct umbilical spread.

Now to sum up. Here we have a child with a widespread mixed infection in the lung, yielding both a haemolytic streptococcus and *Pseudomonas*; the *Pseudomonas* appears to have given rise to a septicaemia involving the liver and the kidney. A swab from the splenic pulp grew a pure culture of *Pseudomonas* which is presumptive evidence of a septicaemia. I did not take a post-mortem blood culture. The size of the lung abscess is remarkable, considering the child lived for only 56 hours. This raises the possibility of infection in utero or at least during birth. The mother's membranes ruptured before she was admitted to hospital so this is possible. On the question of the lack of inflammatory response in the liver and kidneys and the predominantly mononuclear exudate in the lungs, I believe that this child's infection was so overwhelming as to depress the white cell count rather than give rise to the usual leucocytosis. Neuman pointed out in 1890, in a case of systemic *Pseudomonas* infection, this absence of an inflammatory response around bacilli in the liver and spleen.

*Dr. Lloyd:* That poses one or two other problems. There is this question of mixed infections. When did each of them take place and by what route? There is the question of whether the lung abscesses could have taken place as a result of an infection which began not more than 56 hours beforehand. We can discuss that first and see what kind of conclusion we come to. Then there is the fact that the *Pseudomonas* does seem to produce a considerable amount of necrotizing toxin.

I think it would be a good idea, Professor Gillespie, if you were to tell us something about the natural history of these things and which you think contributed more to which lesions in the different parts of the body and in the lung in particular, where we have all this necrosis and abscess formation.

*Professor Gillespie:* What I have to say is mainly about the epidemiological background. This case was in fact one incident in a continuous epidemic involving all the premature babies.

*Dr. Lloyd:* Would you tell us about that then?

*Professor Gillespie:* About a month beforehand another baby had died with cerebral symptoms, and at post mortem (P.M. No. 8707) a similar organism was isolated. At the time this was thought to be an isolated case and nothing much was done to investigate the environment. However, when this second case occurred, we realized that the organism had spread silently through the unit where usually there are about 15 premature babies. So Dr. K. B. Linton set to work and established the source of the organism, where it was breeding, the reservoirs where it was being maintained, and the routes and vehicles which were spreading it amongst the babies. Swabs were taken from mouth, umbilicus and bowel of every baby in the unit. All the babies yielded a similar *Pseudomonas* in one or more sites, and most of the growths were heavy. But no babies were ill except the one we are discussing and the one which died a month before. These organisms were kindly typed for us by Dr. M. T. Parker of the Central Public Health Laboratory, Colindale, using three methods (serological, bacteriophage, and pyocine techniques).

At the same time we cultured the faeces of members of the unit staff, but none of them contained *Pseudomonas*. Twenty babies in other parts of the hospital were cultured and one grew *Pseudomonas* from the bowel but it was of a quite different phage type. Swabs were taken from the furniture, cots, sinks, and various places in the ward, and two *Pseudomonas* cultures were obtained, one from a crack by a sink, a common place for *Pseudomonas*, but this again was a different strain. Settle-plates exposed to the air were all negative.

When investigating *Pseudomonas* infections we must always take cultures from antiseptics and lotions in routine use. Some antiseptic solutions are ineffective against *Pseudomonas*, and may harbour large numbers, and even be reservoirs of them. This was so in our epidemic. One bottle of chloroxylenol (Dettol) and two of a quatenary ammonium detergent (Roccal) contained numerous *Pseudomonas*. The strain in the chloroxylenol was a different type, but those from the detergent bottles were the same type as the epidemic strain. The detergent was used to cleanse babies' skin and could easily transmit the organisms. So to sum up the epidemiology, we have two babies with clinical infections by *Pseudomonas aeruginosa* (*pyocyanea*), but all the other babies in the nursery were "silently" harbouring the same strain. We cannot say where it came from originally, but by the time we investigated it, it was being spread from baby to baby. The spread of *Pseudomonas* was encouraged by the prematurity of the hosts and by the detergent lotion which was acting as its reservoir.

Perhaps I should mention what was done to stop the epidemic. The various vehicles of spread were dealt with by strict barrier-nursing, including the very lavish use of gowns, disposable gloves and hand-washing with 1/5,000 chlorhexidine. The lotion bottles were removed. The principal sources where the organisms were proliferating, the intestinal canals of the infants, were dealt with by giving oral Colistine to all infants. This was surprisingly successful, and faecal cultures from all but two or three infants became negative for *Pseudomonas* in a few days, though coliform bacilli, which were equally sensitive in vitro, persisted apparently undiminished. The umbilical areas were sprayed with a polymyxin preparation.

A final piece of evidence was obtained through a misunderstanding and proved the reservoir role of the detergent. One of the "Roccal" bottles was used again a couple of weeks later and started another wave of "silent" infection, or colonization, by the same *Pseudomonas* strain. No harm was done this time, the bottle was quickly and finally removed, and the outbreak brought to a stop by the same methods as before.

That covers the epidemiology, but there is the question of the other organism.

*Dr. Lloyd:* Yes, the *Pseudomonas* has been causing septicaemia here and has been producing this focal necrosis in the liver; do you suppose it was responsible for all the necrosis and abscess formation in the lungs as well, or is the streptococcus partly responsible for that?

*Professor Gillespie:* I think that the streptococcus was partly responsible. There was a uterine or perinatal infection by the streptococcus and then came the *Pseudomonas* soon after birth.

*Dr. Lloyd:* This accounts for the fact that this baby suffered from *Pseudomonas* infection whereas all the others did not.

*Professor Gillespie:* It is a possibility, I suppose. I want to know whether the morbid anatomists can tell us how long it takes for this sort of abscess to form.

*Dr. Lloyd:* We cannot. We assume they occur not earlier than birth and say "Isn't it wonderful that they could have become so bad in such a short time?"

*Professor Gillespie:* The necrotizing effect is a feature of Gram-negative infection. Endo-toxins in the bloodstream lead to shock and a sort of necrotising effect not only with *Pseudomonas* but other Gram-negative organisms.

*Dr. Corner:* There is another interesting finding. The mother attended ante-natal clinic on 16th July, 6 days before she was delivered and 5 days before going into labour, and she then complained of vaginal discharge. A swab was taken and this was cultured but only *Trichomonas* was found, so that no haemolytic streptococci were isolated 5 days before she went in labour.

*Dr. Lloyd:* If I remember rightly, about 20 or 30 years ago people were culturing disinfectants and finding all sorts of things growing in them, even Dettol was accused of harbouring organisms of one sort or another. If I am right, why is it that "Dettol" is still used so extensively as a disinfectant when it is known to allow the growth of things like *Pseudomonas*?

*Professor Gillespie:* Dettol, and nowadays also the quaternary ammonium compounds, are used as much for their cleansing as for their disinfectant properties. More potent disinfectants are likely to damage a baby's skin.

*Dr. Corner:* The interesting point in the epidemiology is the question of the previous infections that we have had in the unit. We had, last spring, quite a nasty sticky eye which grew *Pseudomonas* but I don't know if that was the same strain.

*Professor Gillespie:* No, that was a different strain.

*Dr. Corner:* Then we had this other baby which died 3 weeks earlier. It is interesting because the mother was unmarried, had no ante-natal care, went into premature labour at home and the baby was brought into us after birth, not many hours old. It died at 3 weeks. We rather assumed that this baby brought the infection in, but it may have picked it up in the unit. At any rate, it seems to have been heavily infected and it looks as though possibly that child was the reservoir from which the infections burst upon us.

*Professor Gillespie:* Of course our experience does show the futility of investigating epidemics merely on clinical grounds. It is no use confining your attention to patients with clinical signs of infection.

*Dr. Corner:* This baby was of course nursed in the same room where the previous baby had been nursed a fortnight before. Some question did arise as to whether it was even in the same incubator.

*Professor Gillespie:* We could not isolate the organism from the incubator.

*Question:* Do you think that these disinfectants are still being used?

*Professor Gillespie:* Definitely not.

*Same voice:* Is that just in this hospital group?

*Professor Gillespie:* Just in this hospital group. I am not sure about other hospitals.

*Dr. Corner:* I think the point about "Dettol" is that it became the fashion when the haemolytic streptococcus was the only organism anybody ever troubled about in maternity hospitals. In the days when we had puerperal sepsis, haemolytic streptococcus was the only organism to be considered. I think I am right in saying that "Dettol" was a very good disinfectant against that organism?

*Professor Gillespie:* Oh, yes. In Colebrook's great work on puerperal sepsis he showed that chloroxylenol could be used in a strength that would kill it and not damage tissue, and it was a great advantage at that time.

*Dr. Corner:* The point about the use of "Roccal" should be made; this is not used as a disinfectant in the ordinary sense but it is one of the best substances for keeping down the urease-producing organisms from the bowel and is used solely for the purpose of swabbing the babies' buttocks to prevent ammonia napkin dermatitis.

*Professor Gillespie:* It is more a cleaning agent than a disinfectant.

*Dr. Lloyd:* Do you use the same bowl of fluid for washing one baby's bottom as you do for washing another's?

*Dr. Corner:* No. Each baby has its own jar of fluid and its own box of swabs and they are all kept separately. Of course there is a stock lotion bottle, and this stock bottle may become infected.

*Professor Gillespie:* A chlorhexidine detergent mixture is now being used.

*Question:* What are thermometers now being cleaned in?

*Professor Gillespie:* They are kept in chlorhexidine.

*Dr. Harbison:* Can I raise two matters. One on Dr. Lloyd's question about the lack of inflammation on these necrotic lesions. Pyocyanin has been demonstrated in vitro to have a toxic effect on leucocytes.

The other point, Professor Gillespie, is that Hoffman and Finburg referring to the mist which is used in incubators, state that *Pseudomonas* can grow in it.

*Dr. Lloyd:* I was going to ask the dermatologists, since we have a great number of them this afternoon, whether they can tell us anything about this particular rash. Is this the kind of rash you expect to get in acute infections or is it peculiar to any one kind of organism like this one?

*Dr. Margaret Walshe:* I should think it could be associated with any severe infection but if it was petechial to start with it would be typical of the rash you get in septicaemia. If you have larger clumps of organisms cast off you get bigger lesions, some of which do break down and become septic.

*Dr. Lloyd:* Yes, this one did, I think.

*Professor Gillespie:* Was there a portal of entry of this organism in this particular baby? Do those findings not suggest that it was through the liver, by the umbilical channel?

*Dr. Harbison:* It is possible; the stump was not running, there was no sepsis around the stump of the umbilical cord when I did the post mortem. This rash was on the abdomen and *Pseudomonas* is know to be able to get through the intact skin.

*Professor Gillespie:* Was there no indication that this had occurred?

*Dr. Corner:* No, but the child had an umbilical catheter for the intravenous glucose drip.

*Professor Gillespie:* But was that not after it became ill?

*Dr. Corner:* Yes, that was after it became ill.

*Professor Gillespie:* But that catheter was not likely to be the portal of entry, unless for the streptococcus.

*Dr. Corner:* Well, that is possible, of course, the drip was set up 26 hours before death, but the baby was pretty bad by that time. On the other hand the blood culture was taken before the drip was set up.

*Dr. Norman Brown:* The only thing against that is these great big abscesses in the lungs. I do not think you can get abscesses like that with ordinary streptococcal infection and I have never seen abscesses as big as that in a newborn child so they must be associated with the *Pseudomonas* infection.

Dr. Harbison pointed out the absence of polymorphs in the inflammatory exudate. I do not think that is surprising because the newborn baby does not react with a polymorphonuclear reaction; it reacts with a mononuclear reaction, and if you see polymorphs in any numbers in newborn baby's lungs it means almost certainly that the pneumonia is a "congenital" one. These are not then the baby's polymorphs but the mother's polymorphs which have been inhaled with the infected amniotic fluid.

